# Ethics of open access to biomedical research: Just a special case of ethics of open access to research

**DOI:** 10.1186/1747-5341-2-31

**Published:** 2007-12-07

**Authors:** Stevan Harnad

**Affiliations:** 1 Institut des sciences cognitives, Département de psychologie, Université du Quebec à Montréal, Montréal, Québec, H3C 3P8, Canada; 2 Department of Electronics and Computer Science, University of Southampton, Highfield, Southampton SO17 1BJ, UK

## Abstract

The ethical case for Open Access (OA) (free online access) to research findings is especially salient when it is public health that is being compromised by needless access restrictions. But the ethical imperative for OA is far more general: It applies to all scientific and scholarly research findings published in peer-reviewed journals. And peer-to-peer access is far more important than direct public access. Most research is funded so as to be conducted and published, by researchers, in order to be taken up, used, and built upon in further research and applications, again by researchers (pure and applied, including practitioners), for the benefit of the public that funded it – not in order to generate revenue for the peer-reviewed journal publishing industry (nor even because there is a burning public desire to read much of it). Hence OA needs to be mandated, by researchers' institutions and funders, for all research.

## 

**1. Research Usage**. All peer-reviewed research articles are written for the purpose of being accessed, used, applied and built upon by all their potential users, everywhere, not in order to generate royalty income for their author (or their publisher). (This is *not *true of writing in general, such as newspaper and magazine articles by journalists, or books. It is only true, without exception, of peer-reviewed research journal articles, and it is true in all disciplines, without exception: [1]).

**2. Publish or Perish**. Research productivity and progress – and hence researchers' careers, salary, research funding, reputation, and prizes – all depend on the usage and application of their research findings ("research impact"). This is enshrined in the academic mandate to "publish or perish," and in the reward system of academic research [2-4].

**3. Access and Impact**. The reason the academic reward system is set up that way is that that is also how research institutions and research funders benefit from the research output they produce and fund: by maximizing its uptake and impact [5]. That is also how the cumulative research cycle itself progresses and grows, along with the benefits it provides to society, the public that funds it [6]: *In order to be fully used, applied, and built upon, research needs to be accessible to all its potential users *(and not only to those that can afford access to the journals in which the research happens to be published).

**4. OA Impact Advantage**. Open Access (OA) – free online access – has been demonstrated [7] to increase research usage and impact by 25%–250% or more [8-14] (Figure 1).

**Figure 1:**
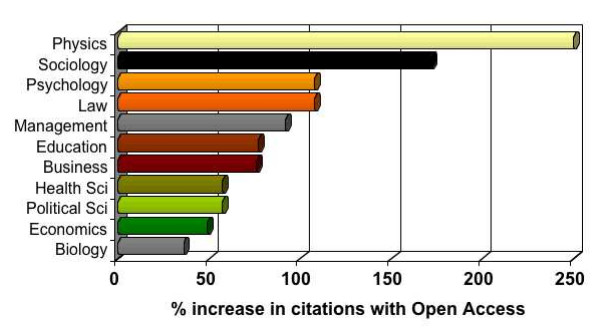
Average citation ratios for articles in the same journal and year that were and were not made OA by author self-archiving. Date span: 1992–2003 [10,11].

**5. All Disciplines**. This "OA Advantage" has been found in all fields: natural sciences, biomedical sciences, engineering, social sciences, and humanities. It can be interpreted as an indication of how much potential research impact is being lost and how much potential access and usage is being denied by not making the research OA. Hence it is true, without exception, in all fields, that the potential research benefit is there, if only the research is made OA.

**6. Online Only**. OA has only become possible since the onset of the online era.

**7. Gold and Green OA**. Research can be made OA in two ways:

**(7a) OA Publishing**. Research can be made "Gold OA" [15,16] by publishing it in an OA journal [17] that makes it free online. (Some OA journals, but not all, cover their costs by charging the author-institution for publishing the article rather than by charging the user-institution for accessing it, but many Gold OA journals today still continue to cover their costs via subscriptions to the paper edition [17].)

**(7b) OA Self-Archiving**. Research can be made "Green OA" [15] by publishing it in a conventional, non-OA journal, but also self-archiving [18] it in the author's Institutional [19] or Central Repository [20], free for all [19,21,22].

**8. Spontaneous OA**. Despite its benefits to research, researchers (pure and applied), their institutions, their funders, the R&D industry, practitioners [23], and the tax-paying public that funds the research, only about 15% of the approximately 2.5 million peer-reviewed research journal articles published every year are being spontaneously self-archived by their authors today (Green OA) [24]. A somewhat lower percentage of articles are being published in Gold OA journals, deterred in part by the cost. Of the approximately 25,000 [25] peer reviewed journals published today, about 10% [17] are OA. A recent survey of University of California faculty found that 21% had published at least one article in an OA journal, whereas 31% had self-archived at least one article on their website and 14% in their Institutional Repository; 29% had self-archived at least one preprint of an article [26].

**9. Mandated OA**. Only Green OA is entirely within the hands of the research community. *Researchers' funders and institutions cannot mandate Gold OA (hence should not waste time trying*): Research funders and universities cannot require the non-OA publishers of the 90% of journals that are non-OA to convert to OA publishing; nor can they require their researchers to pay OA publishing charges when the potential funds to pay for it are still tied up in paying for non-OA subscriptions; nor can (or should) researchers' funders and institutions divert funds from elsewhere to pay the extra cost of OA publishing today; nor can (or should) researchers' funders and institutions try to decide for their researchers what is the best journal for their research to be reported in.

**10. Self-Archive to Flourish**. *But researchers' funders and institutions can (and hence should – and indeed more and more already do) mandate *[27]*Green OA self-archiving*, as a natural extension of their existing "publish or perish" mandate, to maximize the usage and impact of their research in the online era ("self-archive to flourish"). A growing number of institutions and funders are now beginning to adopt Green OA mandates, especially in the UK, and also in Europe and Australia. The US is only beginning to propose mandates; it adopted a Green OA mandate to self-archive NIH-funded research in December 2007. To date, a total of 35 Green OA mandates has been adopted, and 8 more proposed [28].

**11. Green Journals**. About 62% of journals [29] are Green, meaning they already formally endorse the OA self-archiving of the author's peer-reviewed final draft immediately upon acceptance for publication. This means that 62% (rather than 15%) of articles could already be made immediately OA today. In addition, 38% of journals either endorse an embargo of 6 months or longer before the article is made OA or they do not endorse making it OA at all [30]. There is a solution even for this 38%, however:

**12. Mandatory Deposit, Delayed OA**. All the mandates can already require immediate deposit in all cases, specifying that access-privileges for any embargoed deposits may be made *Closed Access *rather than Open Access during the embargo [27]. This means that only their bibliographic metadata are accessible directly; but the Institutional Repositories have an email address box plus a semi-automatized "email eprint request" button that would-be users can press to request an individual copy for research purposes [31]. The author receives the email immediately and need merely click to authorize the immediate automatic emailing of the eprint to the requester [31]. So Green OA self-archiving mandates can already provide immediate OA for 62% of articles, and almost-immediate, almost-OA for the remaining 38%.

**13. Conversion to Gold**. Some publishers are lobbying against Green OA self-archiving mandates, claiming they will destroy peer review and publishing [32]. All existing evidence, however, is contrary to this [22,33]. (In the few fields where Green OA already reached 100% some years ago, the journal subscriptions are still not being cancelled.) Moreover, it is quite clear that even if and when 100% Green OA should ever lead to unsustainable subscription cancellations, journals can and will simply convert to Gold OA and institutions will then cover their own outgoing Gold OA publishing costs by redirecting part of their windfall subscription cancellation savings on incoming journal articles (published by other institutions) to cover instead the Gold OA publishing costs for their own institution's journal article output [34]. The net cost will also be much lower, as it will only need to pay for peer review [35] and its certification by the journal's name and track-record, as the distributed network of OA Institutional Repositories will be the online access-providers and archivers (and the paper edition will be obsolete) [36].

**14. Taxpayer Access**. One of the ways the OA movement is countering the lobbying of publishers against Green OA mandates is by forming the "Alliance for Taxpayer Access" (ATA) [37]. This lobbying group is currently focusing mainly on biomedicine, and the potential health benefits of tax-payer access to biomedical research. This is certainly a valid ethical and practical rationale for OA, but it is definitely not the sole rationale, nor the primary one [38]. (ATA does stand ready to lobby in support of the full spectrum of research, for example, if the Federal Research Public Access Act is reintroduced in the US Congress [39]. But ATA's rationale relies heavily on public access, rather than researcher-to-researcher access.)

**15. Researcher Access**. The primary, fundamental and universal rationale for OA and OA mandates, in all disciplines, including biomedicine [40], is *researcher-to-researcher access *(including pure and applied researchers, as well as practitioners, if any; [23]), not *public access *(nor even educational access). The vast majority of peer-reviewed research in all disciplines is not of direct interest to the lay public (nor even to students, other than graduate students, who are already researchers). And even in biomedical research, what provides the greatest public benefit is the potential research progress (leading to eventual applications and cures that benefit the public) that results from maximizing researcher-to-researcher access. *Direct public access of course comes with the OA territory, but that is not the sole or primary ethical justification for OA, even in biomedical research*.

**16. The Gutenberg Era**. The general ethical rationale and justification for OA is that research is funded, conducted and published (i) in order to be used and applied as widely as possible, not (ii) in order to generate revenue for the journal publishing industry. In the Gutenberg era of paper publication, the only way to achieve the former was by allowing access to be restricted to those researchers whose institutions could afford to subscribe to the paper edition. That was the only way the true and sizeable costs of peer-reviewed research publishing could be covered at all, then.

**17. The PostGutenberg Era**. But in the PostGutenberg era of online communication this is no longer true. Hence it is time for the institutions and funders who employ the researchers and fund the research to mandate that the resulting journal articles be made (Green) OA, to the benefit of the entire research community, the vast R&D industry, and the tax-paying public. (This may or may not eventually lead to a transition to Gold OA; [36,41].)

**18. Maximizing Public Good**. It is unethical for the publishing tail to be allowed to continue to wag the research dog. The dysfunctionality of the status quo is especially apparent when it is public health that is being compromised by needless access restrictions, but the situation is much the same for all scientific and technological research, and for scholarship too, inasmuch as we see and fund scholarly research as a public good, not as a subsidy to the peer-reviewed journal industry [42].

## Competing interests

The author(s) declare that they have no competing interests.
